# A Review on the Present Advances on Studies of Toxoplasmosis in Eastern Africa

**DOI:** 10.1155/2020/7135268

**Published:** 2020-07-06

**Authors:** John Mokua Mose, John Maina Kagira, David Muchina Kamau, Naomi Wangari Maina, Maina Ngotho, Simon Muturi Karanja

**Affiliations:** ^1^Department of Medical Laboratory Sciences, School of Health and Biomedical Sciences, The Technical University of Kenya, Nairobi, Kenya; ^2^Department of Animal Health and Production, College of Agriculture and Natural Resources, Jomo Kenyatta University of Agriculture and Technology, Nairobi, Kenya; ^3^Department of Public Health, Jomo Kenyatta University of Agriculture and Technology, College of Health Sciences, Nairobi, Kenya; ^4^Department of Biochemistry, College of Health Sciences, Jomo Kenyatta University of Agriculture and Technology, Nairobi, Kenya; ^5^Department of Animal Health & Production, Mount Kenya University, Thika, Kenya

## Abstract

Toxoplasmosis is a zoonotic infection caused by the protozoan parasite, *Toxoplasma gondii*. It was discovered over 100 years ago and is credited as the most successful parasitic organism worldwide, able to infect and multiply in all warm blooded animals including an estimated 2.3 billion people. Toxoplasmosis is asymptomatic in immunocompetent individuals. Infection in the developing fetus and immunocompromised individuals can cause severe clinical disease. Toxoplasmosis is also a major cause of reproductive failure in livestock. The economic impact of toxoplasmosis is believed to be substantial. Factors associated with toxoplasmosis infection have been defined. Eastern Africa region is a high-risk area mainly due to the close association of humans and livestock as well as sociocultural practices, poor environmental hygiene, and poverty. The present paper provides a narrative review of published data on toxoplasmosis in Eastern Africa.

## 1. Scope of Review

**Figure 1:**
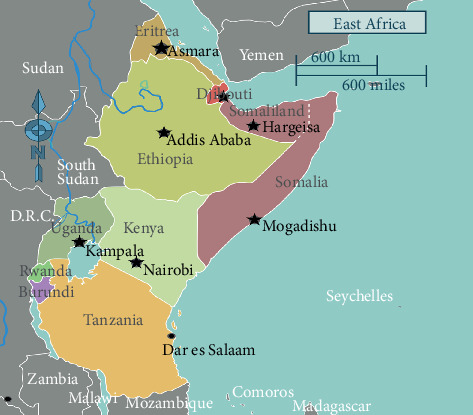
Location of countries constituting Eastern Africa (https://commons.wikimedia.org/wiki/File:East_Africa_regions_map.png).

Eastern Africa is the easterly region of the African continent constituting 11 countries, namely, Kenya, Tanzania, Uganda, Rwanda, Burundi, Ethiopia, Eritrea, Somalia, Djibouti, The Sudan (Khartoum), and South Sudan (Juba) (Figure 1). The estimated population size of Eastern Africa is 290 million (http://www.afdb.org/en/countries/east-africa/). Economically, these countries are ranked either as developing or underdeveloped. The present paper reviews the literature on *Toxoplasma gondii* infection in humans and animals in Eastern Africa. For the purposes of this review, literature on Sudan will be taken to include both The Sudan (Khartoum) and South Sudan (Juba). All retrieved studies were studied carefully, and the extracted data included the year of publication, characteristics of the study population, location of the study, sample size, and number of cases. Reference lists of full-text publications and textbooks were also examined to identify studies not retrieved by the original search.

## 2. Introduction

Toxoplasmosis is a neglected tropical disease of poverty caused by the obligated intracellular protozoan parasite, *Toxoplasma gondii* [1, 2]. This facultative heterogeneous parasite belongs to the phylum Apicomplexa, class Conoidasida, subclass Coccidiasina, order Eucoccidiorida, family Sarcocystidae, genus *Toxoplasma*, and species *gondii*.

*Toxoplasma gondii* is a well-studied parasite because of its medical and veterinary importance and its suitability as a model for cell biology and molecular studies with a unicellular organism [3]. The name *Toxoplasma gondii* is derived from the Greek word (toxon: bow and plasma: shape), whereas *gondii* is derived from the rodent *Ctenodactylus gundi* from which it was first isolated in 1908.

The parasite is a cosmopolitan protozoon with no host specificity in the asexual stage (it can parasitize all mammals, including humans and felids), whereas in the sexual stage it is specific to felids [4]. The wide range of warm-blooded hosts, including infection of one-third of the global human population, makes *T. gondii* the most successful parasitic organisms worldwide.

**Figure 2:**
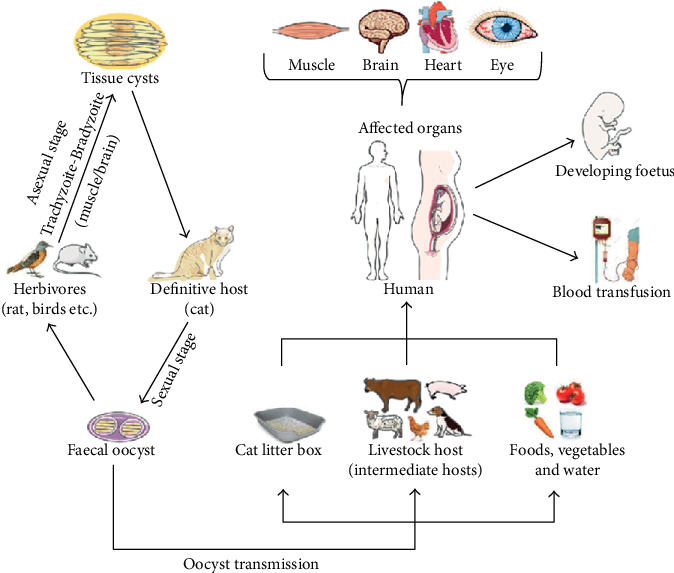
Transmission dynamics of *Toxoplasma gondii* [2].

Transmission of *T. gondii* is multifaceted. Firstly, it can be transmitted from definitive to intermediate hosts and vice versa. Secondly, the parasite is transmissible between definitive hosts. Thirdly, transmission of *T. gondii* can occur between intermediate hosts (Figure 2). Human infection may be acquired in several ways: ingestion of undercooked infected meat containing *T. gondii* cysts; ingestion of the oocyst from faecally contaminated hands, food or water; organ transplantation or blood transfusion; transplacental transmission; and accidental inoculation of tachyzoites [5].

### 2.1. Clinical Disease

The clinical manifestations of toxoplasmosis vary depending on parasite characteristics such as virulence of the strain and inoculum size, as well as host factors such as genetic back ground and immune status [6]. There are at least three genetic types of *T. gondii*: types I, II, and III. They differ in virulence and epidemiological pattern of occurrence [7]. Genetic diversity of *T. gondii* is tackled later in this review.

In animals, *Toxoplasma gondii* can cause subclinical infections or clinical disease with a wide range of clinical signs in intermediate or definitive hosts. High prevalence of toxoplasmosis in domestic and wild animals throughout the world has been documented [8]. Seropositivity in food-producing animals is of veterinary and medical health problem because it represents a real risk for transmission of the disease to humans, either directly or through farming. Pigs, cattle, poultry, sheep, goats, and horses are major reservoirs for human infection [9].

Epidemiological studies on toxoplasmosis in sub-Saharan Africa are scarce despite its multifaceted yet easy transmission dynamics [10]. Generally, it is known that ingestion of undercooked meat containing *T. gondii* tissue cysts, especially from pigs, lambs, goats, and chicken, or consumption of food and water contaminated with oocysts from cat feces is the most common route for human infection.

The likely sources of infection for pigs include ingestion of feed contaminated with cat feces, eating infected rodents, and cannibalism. Pigs and other small stock are often slaughtered in unhygienic conditions which may increase transmission of zoonoses, including toxoplasmosis [11]. Chicken can also be infected by *T. gondii* and act as a source of infection for humans. Free-range chicken becomes infected mostly by feeding from grounds contaminated with oocysts, and hence, the prevalence of *T. gondii* in chickens is a good indicator of the type of strains and oocyst burden in the environment [12, 13].

Toxoplasmosis infection in livestock leads to significant economic losses as a result of reproductive failure, i.e., abortion, fetal resorption, and barrenness. Fortunately, recent studies indicate that the prevalence of *T. gondii* in meat-producing animals decreased considerably over the past 20 years in areas with intensive farm management [5]

### 2.2. Infections in Humans

Disease in humans caused by *T. gondii* was first recognized in the late 1930s [6]. Improved diagnostic techniques continue to enable seroepidemiological studies in humans as well as a broad range of animal species which provides evidence for a wide distribution and high prevalence of *T. gondii* in many areas of the world. It has been estimated that up to one-third of the world human population has been exposed to the parasite [14]. However, seroprevalence estimates for human populations vary greatly among different countries, among different geographical areas within one country, and among different ethnic groups living in the same area. Thus, over the past 3 decades, antibodies to *T. gondii* have been detected from 0 to 100% of individuals in various adult human populations.

The global picture of toxoplasmosis presents a grim image. In the Americas, the prevalence of *T. gondii* ranges from 16% in North America to 75% in parts of Middle and South America. The disease disproportionately affects poor minority populations in United States of America. Europe has also been observed to have highly variable prevalence ranging from 10% in Iceland to 63% in Poland [15].

Neglected tropical zoonotic diseases are highly endemic but patchily distributed among the 20 countries and almost 400 million people of the Middle East and North Africa (MENA) region. However, there is scarce information on the prevalence of toxoplasmosis in these countries. Some data shows that the disease is highly prevalent in women of child bearing age and HIV-positive patients [16]. Few studies have been done on human population in Australia and New Zealand showing prevalence of up to 50%. Extensive epidemiological studies have been conducted in China and India revealing high prevalent rates in human populations [17].

The study of toxoplasmosis has also been neglected in sub-Saharan Africa (SSA), although investigations conducted in several countries indicate high prevalence [10]. In fact, a search through published data on SSA reveals prevalence rates of > 40% in most populations surveyed [15]. The bulk of studies has been conducted in Nigeria and Southern Africa.

### 2.3. Clinical Disease in Humans

**Figure 3:**
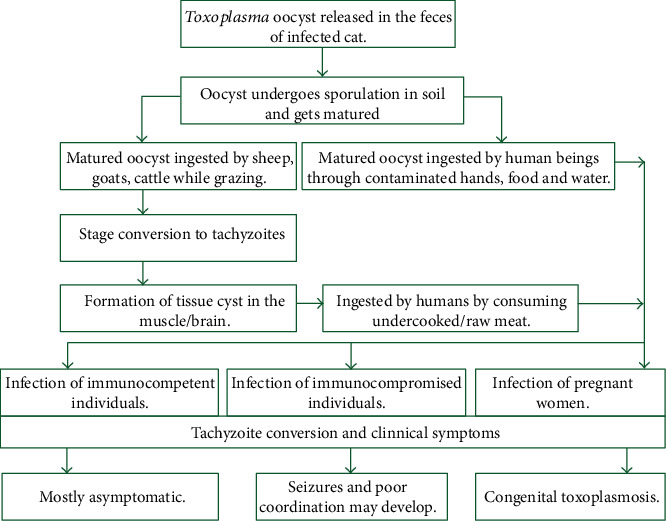
Illustration summarizing the relationship between transmission and clinical manifestations of toxoplasmosis [18].

*Toxoplasma gondii* infects a large proportion of the world's population but rarely causes clinically significant disease. Asymptomatic infection with *T. gondii* resulting in a latent infection with tissue cysts is common in humans (Figure 3; [18]). However, certain individuals are at high risk for severe or life-threatening toxoplasmosis. These individuals include fetuses, newborns, and immunologically impaired patients where *T. gondii* can cause several clinical syndromes including encephalitis, chorioretinitis, congenital infection, and neonatal mortality [6] and postnatally acquired toxoplasmosis in immunocompetent humans.

While infection with *T. gondii* in humans is very common, clinical disease is largely confined to risk groups [6]. Most cases of *T. gondii* infections in immunocompetent humans are asymptomatic. Occasionally, mild symptoms may be observed characterized by lymphadenopathy, the most significant clinical manifestation. Severe manifestations, such as encephalitis, sepsis syndrome/shock, myocarditis, or hepatitis, may occur but are very rare in immunocompetent humans.

Infection with *T. gondii* has also been recognized as an important cause of retinochoroiditis. Ocular toxoplasmosis has long been regarded as a result of a prenatal infection with *T. gondii*, which manifests later in life. However, there are now several recorded cases in which the development of ocular symptoms, such as retinitis and retinochoroiditis, was convincingly associated with acquired toxoplasmosis in humans. Thus, it has now become clear that ocular toxoplasmosis may be both a result of a prenatal infection or an infection acquired postnatally [19].

### 2.4. Congenital Toxoplasmosis

If a primary *T. gondii* infection is acquired 4 ± 6 months before conception or earlier, protective immunity will usually prevent vertical transmission to the fetus on subsequent exposures. However, if first contracted during pregnancy, *T. gondii* may be transmitted to the fetus in immunocompetent women [20]. The mechanism of vertical transmission is not yet understood. A probable scenario is that temporary parasitaemia in a primarily infected pregnant woman may result in the invasion of the placenta by tachyzoites which then multiply within cells of the placenta. Eventually, some of these cross the placenta and enter the fetal circulation or fetal tissues [21]. Congenital toxoplasmosis may cause abortion, neonatal death, or fetal abnormalities with detrimental consequences for the fetus. It may also significantly reduce the quality of life in children who survive a prenatal infection [15].

The risk of intrauterine infection of the fetus, the risk of manifestation of congenital toxoplasmosis, and the severity of the disease depend on the time of maternal infection during pregnancy, the immunological competence of the mother during parasitaemia, the number and virulence of the parasites transmitted to the fetus, and the age of the fetus at the time of transmission [22]. If not treated, the risk of intrauterine infection of the fetus increases during pregnancy, i.e., from about 14% after primary maternal infection in the first trimester to about 59% after primary maternal infection in the last. While the risk of intrauterine infection of the fetus increases during pregnancy, the effects on the fetus are more severe if transmission occurs at an early stage of pregnancy [21].

The most significant manifestation in the fetus is encephalomyelitis which may have severe consequences, abortion and neonatal deaths. Signs of the classic triad of toxoplasmosis (retinochoroiditis, intracranial calcifications, and hydrocephalus) manifest in newborns [6]. Other newborns show a variety of symptoms, ranging from visual and central nervous symptoms to nonspecific symptoms of acute infection (retinochoroiditis, convulsions, splenomegaly, hepatomegaly, fever, anaemia, jaundice, lymphadenopathy, etc.). About 12 ± 16% of these newborns die from the disease [15]. The surviving infants may suffer from progressive visual and mental retardation or other neurological deficiencies which often require special education and residential care [23].

### 2.5. Toxoplasmosis in Immunocompromised Individuals

Pregnant and immunocompromised individuals have a significantly higher chance of *T. gondii* infection than the immunocompetent [24]. In immunocompromised humans a previously acquired latent infection can lead to reactivated toxoplasmosis with encephalitis. Toxoplasmic encephalitis and disseminated toxoplasmosis have been observed in patients with immunodeficiencies due to various causes, such as Hodgkin's disease or immunosuppressive therapy because of other malignancies [25]. Disseminated toxoplasmosis may also complicate transplantation of organs or bone marrow. This may result either from transplantation of an organ from a *T. gondii*-infected donor to a susceptible recipient or from reactivation of a latent *T. gondii* infection in the recipient due to immunosuppressive treatment [26].

*Toxoplasma gondii* is an important opportunistic pathogen in AIDS patients causing severe encephalitis and death in over 30% of these patients. Administration of highly active antiretroviral therapy (HAART) and immune reconstitution reduces the incidence of CNS toxoplasmosis in AIDS patients which is now declining in many countries. Reactivation of a latent infection can also be prevented by prophylaxis with trimethoprim-sulfamethoxazole (TMX-Sulfa). In addition to reactivated toxoplasmosis, immunocompromised patients are at risk from severe disease following primary infection, which frequently presents as pulmonary disease or diffuse encephalitis [27].

### 2.6. Risk Factors for *T. gondii* Infection in Humans

Infection in humans is mainly acquired by oral ingestion of food or water that is contaminated with oocysts shed by cats or by eating undercooked or raw meat containing tissue cysts [12, 28]. Classically, consumption of undercooked meat, particularly pork and lamb, has been ascribed to be the major risk factor for acquisition of toxoplasmosis.

Variations in seroprevalence of *T. gondii* seem to correlate with eating and hygiene habits of a population. Improved animal husbandry practices as well as increased awareness of the risks of consuming undercooked meat have resulted in decreased prevalence of toxoplasmosis [9]. Transmission of *T gondii* by organ transplantation from a seropositive donor to a seronegative recipient (donor (D) +/recipient (R) –) is an important potential cause of disease in heart, heart-lung, kidney, liver, and liver-pancreas transplant patients [26]. The recognition of waterborne toxoplasmosis in humans has provided another dimension to the epidemiology of this infection [29]. Transmission during breastfeeding or direct human-to-human transmission other than from mother to fetus has not been recorded.

### 2.7. Diagnosis

An accurate diagnosis of toxoplasmosis constitutes an important measure for the control of the disease, particularly during pregnancy. It may also avoid serious economic losses in the sheep and goat industry. *Toxoplasma gondii* infection can be diagnosed using direct or indirect techniques [14].

Indirect serological techniques serological diagnosis entails detection of specific anti-*Toxoplasma* immunoglobulins, i.e., IgM, IgG, or IgA [30]. This is accomplished through application of immunology-based techniques, e.g., enzyme-linked immunosorbent assays (ELISA), Sabin-Feldman dye test, immunofluorescent assay (IFA), or modified agglutination test (MAT).

Direct diagnosis entails the detection of whole or fractions of parasite, e.g., nucleic acids or proteins by polymerase chain reaction (PCR), loop-mediated isothermal amplification (LAMP), hybridisation, and histology. Whereas indirect serological methods are widely used in immunocompetent patients, definitive diagnosis in immunocompromised people is mostly undertaken by direct detection of the parasite. To discriminate chronic from reactivated infection, IgG avidity can also be determined with VIDAS instrument (bioMerieux, France). Common *T. gondii* targets for direct diagnosis are the repetitive 529 base pairs fragment repeated 200–300 times in the parasite genome and B1 gene [31].

### 2.8. Treatment

Sulfonamides, clindamycin, spiramycin, and pyrimethamine are effective against *T. gondii* infection. The drug combinations sulfadiazine/pyrimethamine and sulfadoxine/pyrimethamine have a synergistic anti-Toxoplasma effect. The recommended prophylaxis against toxoplasmosis in immunocompromised patients is through administration of co-trimoxazole (trimethoprim plus sulfamethoxazole or TMP-SMX). Treatment regime entails one double strength or two single strength daily doses for life or until CD4 counts exceed 200 cells/mm^3^ on highly active antiretroviral therapy [32].

### 2.9. Genetic Diversity of *T. gondii*

The genotype of an infecting *T. gondii* strain may determine the outcome of infection and the reactivation risk of chronic disease. *Toxoplasma gondii* consists of three clonal lineages designated types I, II, and III, which differ in virulence and epidemiological pattern of occurrence [33]. Most strains isolated from patients with AIDS are type II. Type I and II strains have been recorded in patients with congenital disease, whereas strains isolated from animals are mostly genotype III. The generation of specific gene-deficient strains of *T. gondii* and sequencing of the *Toxoplasma* genome (http://ToxoDB.org/) will provide further insight into virulence factors of the parasite and specific host immune responses. Sexual recombination between two distinct and competing clonal lines of the parasite has driven natural evolution of virulence in *T. gondii* [34].

A PCR restriction fragment length polymorphism (RFLP) assay was designed to differentiate the three genotypes of *T. gondii*, based on polymorphism of the SAG2 gene locus. Subsequently, numerous high-resolution assays have been developed for typing *T. gondii* ranging from microsatellite analysis to the use of synthetic peptides [35]. While these high-resolution strain-typing techniques provide superior genetic data, they are more labour intensive and the PCR-RFLP-based genotyping is still used for preliminary genotyping isolates [36].

### 2.10. Review of Studies on Toxoplasmosis in Eastern Africa

The following is a country-by-country compilation of information on toxoplasmosis in Kenya, Ethiopia, Uganda, Tanzania, The Sudan, Rwanda, Burundi, Eritrea, Djibouti, and Somalia.

#### 2.10.1. Kenya

Cases of toxoplasmosis have been reported in Kenya. The earliest study was documented in 1968 [37]. Since then, *T. gondii* has been detected in the general Kenyan population as well as susceptible groups with reduced immunity. A serological survey of 127 children revealed a significant rise of prevalence of the *T. gondii*-specific antibodies from 35% in preschool to 60% in the early school age group [38]. The findings suggested that poor sanitary habits and conditions as well as water shortage in primary schools may cause parasitic infection through contact between children.

Screening results for blood donors at Kenyatta National Hospital in Nairobi indicated high seroprevalence [39]. Fifty-four percent (54%) of HIV-positive patients attending Kenyatta National Hospital, Nairobi, had *Toxoplasma*-specific IgG in contrast to 1% of the HIV-negative group [40]. In a similar study carried out at a private teaching hospital in Kenya, a seroprevalence rate of 32% was found [41].

A clinical case report of toxoplasmosis was documented in a patient with HIV infection [42]. Another study done in antenatal women attending Kenyatta National Hospital, Kenya, revealed a high percentage of pregnant women with anti-*T. gondii* either acute (23%) or chronic (30%) seropositivity [43]. About 12.7% of hospitalised HIV-positive patients with neurological complications at a private hospital in Nairobi had *T. gondii* infection [44]. Another study was done involving the development of a neurological mouse model for Toxoplasmosis using *Toxoplasma gondii* isolated from chicken in Kenya. The brain of toxoplasmosis-infected mice showed mononuclear cellular infiltrations, neuronal necrosis, and perivascular cuffing [45].

Coinfection of *T. gondii* and other parasites such as *Toxocara canis* has been investigated using samples from Kenyans. *Toxoplasma gondii* detected in five of seven *T. canis*-positive sera from Maasailand [46]. Such publications and clinical case reports show that there is a widespread distribution of multiple infections involving *T. gondii* in Kenya.

Chunge and colleagues investigated the prevalence of antibodies to *T. gondii* in serum samples from pregnant women and cord blood at Kenyatta National Hospital, Nairobi [47]. This is the only documented study on *T. gondii* infection during pregnancy in Kenya. Natural *T. gondii* infection has been detected in free-living and captive animals [48]. Of these, 8 of 8 (100%) captive carnivores, 14 of 19 (74%) captive herbivores, 11 of 14 (79%) free-living carnivores, and 97 of 118 (82%) free-living herbivores were found to have *Toxoplasma* antibodies.

The detection of *Toxoplasma gondii* in free-range chickens is a good indicator of possible risk to human beings. In a study carried out in Thika region Kenya, the prevalence of *T. gondii* was 79.0% (95% CI: 70.0–86.4%). The results of this study indicated a high level of Toxoplasma infection in free-range chicken in Kenya, and this could indicate environmental contamination with *T. gondii* oocysts [13]. In another study carried out by Njuguna et al. [49] to establish the prevalence of gastrointestinal (GIT) parasites in fecal samples of 103 cats kept by households in Thika region, *Toxoplasma gondii* was detected in 7.8% (95% CI: 4.5–11.1%) of the samples collected. Another recent study in Thika region showed that up to 39% of the slaughterhouse workers were infected with *T. gondii* as detected using nPCR [50].

In another similar study, the biologic and genetic characterization of *T. gondii* isolates from Kenya was conducted on free-ranging chickens [12]. *Toxoplasma gondii* antibodies were found in 4 of 30 chickens, and *T. gondii* was isolated from the brain of 1 of 4 seropositive chickens; this strain was avirulent for mice and was type II. A separate study confirmed that chicken-infective *T. gondii* strain in Kenya is mainly type II [36].

#### 2.10.2. Ethiopia

Ethiopia has the highest number of publications on toxoplasmosis among the Eastern African countries. In a recently published review, Dubey and colleagues showed that there is high prevalence of toxoplasmosis in both humans and animals in Ethiopia [51]. In fact on the basis of a conservative *T. gondii* seroprevalence of 50%, the authors acknowledge that thousands of immunocompromised individuals might die of concurrent opportunistic infections, including toxoplasmosis. However, there is limited data on seroprevalence in pregnant women and congenital toxoplasmosis and immunocompromised individuals.

Few documented publications include studies on high-risk groups of pregnant women and HIV-positive individuals. A community-based cross-sectional study revealed a high eroprevalence of *T. gondii* (83.6%) and significant presence of associated risk factors among pregnant women in Jimma Town, South West Ethiopia [52]. Gebremedhin and colleagues revealed *T. gondii* seroprevalence of 81.4% in women of child-bearing age in central Ethiopia. These studies showed that pregnant women having domestic cats at home were at higher risk of infection. Additionally, there is significant association between illiteracy (or level of education), study area, age, pregnancy status, raw vegetable consumption, source of water, presence of cats at home, contact with cats, HIV status, and precaution during cats' feces cleaning [53].

A comparative cross-sectional study in Addis Ababa revealed that the seroprevalence of latent *T. gondii* infection among the participants was 90.0% (297/330). *Toxoplasma gondii* infection was observed with respective prevalence of 93.3% (154/165) and 86.7% (143/165) among HIV-infected and HIV-uninfected people [54]. However, this study was not specifically designed to study toxoplasmosis and missed data on basic risk factors such as cat ownership, dietary habits, and soil exposure and other important risk factors for disease acquisition. More objective studies on HIV-positive individuals disclosed a seroprevalence of 87.4% (90/103) and 10.7% (11/103) for anti-*T. gondii* IgG and IgM antibodies, respectively [55, 56]. Multivariate analysis using logistic regression showed that anti-*T. gondii* seropositivity was independently significantly associated with undercooked or raw meat consumption, sex (more males than females), and having contact with cat.

Studies have been conducted to explore the hypothesis that symptoms of schizophrenia may be related to infection of the central nervous system with *T. gondii* [53]. Results indicate that 87.9% of participants were positive for *T. gondii* IgG antibody [46]. However, patients in both treatment groups (the trimethoprim and placebo group) did not show significant between-group differences. This lack of significant difference suggests that treatment with trimethoprim is not superior to placebo in improving symptoms of schizophrenia. Therefore, it was not possible to draw firm conclusion(s) regarding the etiological role of toxoplasmosis on schizophrenia based on this study. Adolescents (13 to 26 years old) have also been surveyed where a seroprevalence of 71.43% was reported [57].

Another cross-sectional study was conducted to determine the seroprevalence and potential risk factors of *T. gondii* infection in pregnant women attending antenatal care at Bonga Hospital, Southwestern Ethiopia. Out of the total of 210 pregnant women, 159 (75.7%) were seropositive for *T. gondii*, and the odds ratio of having *T. gondii* infection was high in pregnant women within the age category 36–44 years [58]. A similar study conducted in at Hawassa University comprehensive specialized and Yirgalem General Hospitals in Southern Ethiopia reported a weighted prevalence of 81.8% for the anti-*Toxoplasma gondii* antibody, and a significant association was observed between seroprevalence and contact with domestic cats [59].

Other studies have been conducted in different geographical locations of Ethiopia indicating a high seroprevalence of *Toxoplasma gondii* infection in sheep, goat, and pig. A cross-sectional study was carried out to assess the seroprevalence and identify risk factors of *Toxoplasma gondii* infection in domestic ruminants of East Hararghe zone of Oromia region, Ethiopia. Overall, the prevalence of *T. gondii* infection in domestic ruminants was 22.2% (302/1360). The seroprevalence in sheep, goats, cattle, and camels was 33.7%, 27.6%, 10.7%, and 14.4%, respectively [60]. Another recent study conducted in southern Ethiopia reported an overall seroprevalence of 26.09% *Toxoplasma gondii* antibodies in sheep and goats [61]. In another study conducted in Addis Ababa by Dubey et al. [62], a seropositive rate of 91.6% was reported among the randomly sampled cats.

#### 2.10.3. Uganda

A fair share of data on toxoplasmosis infection from Uganda has been brought to the public domain. In a seroprevalence study conducted as early as 1970, the researchers sought to establish whether there is a relationship between *T. gondii* and *Toxocara canis* infections. Notably, of the 34 *Toxocara*-positive sera from Uganda, six (18%) were positive to the toxoplasma dye test [46]. Results showed that incidence of concurrent infections was no greater than that expected for a normal population. This study showed no causal or clinically important relationship between toxoplasmal and toxocaral infections.

Uganda has one of the highest HIV prevalence rates in sub-Saharan Africa (6.5%) [63]. Unsurprisingly, seroprevalence of toxoplasmosis among HIV-positive Ugandans varies from 34% to 54%. Reactivated infection has been detected in 23% of these patients. Further, almost one in four of these patients who have focal neurology suffers from toxoplasmosis caused by reactivated parasites [64].

In a cross-sectional study carried out in Uganda, the occurrence of porcine *Toxoplasma gondii* infections in smallholder production systems was determined. The sera were tested for the presence of antibodies to *T. gondii*. An overall seroprevalence based on the commercial ELISA was 28.7% (95% CI: 25.8-31.7%). Seropositive animals were found in all villages with significant differences across the three districts (*P* < 0.05) and 12 subcounties (*P* < 0.01) in the survey area [65].

There is limited data on *T. gondii* infection in livestock from Uganda where livestock farming is important to the local economy and also a popular source of food. The level of infection was 31% and 47% in goats and chicken respectively [66]. The high seroprevalence detected in popular livestock suggests that they may play a significant role in zoonotic transmission to humans.

Natural recombination between clonal lineages has been demonstrated using isolates from Uganda. A type II/III recombinant isolate, TgCkUg2, was shown to contain entire chromosomes of either type II or type III origin, demonstrating chromosome sorting rather than intrachromosomal recombination. In Ugandan HIV-positive patients, type II allele is responsible for causing most clinical disease [67]. All three genotypes have been detected in chicken with some of the animals having multiple infections.

#### 2.10.4. Tanzania

The burden of *T. gondii* was studied in the population of Nyamisati village, Tanzania. The seropositivity rate was 4% (19/450) among the subjects of Nyamisati origin and 47% (15/32) among immigrants from other areas of Tanzania [68]. The generally low transmission in this mainly Muslim village appeared to be related to sparse consumption of contaminated food and low prevalence of oocysts due to scarcity of felines. A bigger serological survey carried out in Tanga district of north-eastern Tanzania aimed at assessing *T. gondii* infection rates among occupationally exposed groups including abattoir workers, livestock keepers, and animal health workers [69]. Antibodies to *T. gondii* were detected in 91 (46%) of the 199 individuals studied. The seroprevalence of toxoplasma antibodies was significantly higher among individuals who keep livestock (52.2%) and abattoir workers (46.3%). These results strengthen previous findings that consumption of raw or undercooked meat and keeping pets especially cats presents more of the risk factors than occupational groups.

Concurrent infections involving toxoplasmosis have been documented [46]. *Toxoplasma gondii* was detected in four out of 13 *Toxocara*-positive sera from Dar es Salaam, Tanzania. There is scarce information on toxoplasmosis infection in Tanzanian HIV-positive individuals. A case of a 35-year old HIV-positive man with fulminant toxoplasma encephalitis (TE) was reported [70]. The patient presented with a one week history of severe headache and treated empirically with antimalarial drugs. TE was diagnosed postmortem histologically by haematoxylin-eosin and immunohistochemical stain with P30 antibody for toxoplasma antigen. This case study report proved that TE is ignored leading to misdiagnosis with death being a likely outcome. Neuropathological findings, including TE, were observed in 31 out of 52 HIV-infected patients in a forensic autopsies study [71]. A similar study was carried out to determine the seroprevalence of specific *Toxoplasma gondii* IgG antibodies among HIV/AIDS patients attending Bugando Medical Centre in Mwanza, Tanzania [72].

*T. gondii*-specific IgG antibodies were found in 26 (68.4%) of the patients with immunological failure compared to 46 (32.86%) of those without immunological failure (OR: 4.42, CI: 2.05-9.55; *P* < 0.001). In another study, occupationally exposed individuals were identified with a seroprevalence of 46%, while 35% and 30.9% of pregnant women were seropositive for *T. gondii* antibodies.

Toxoplasmosis was found to have a prevalence rate exceeding 35% in normal pregnant women in Tanzania. However, in pregnant anaemic women and those suffering from hypertension, prevalence was significantly higher at 52.5% and 66.7% respectively [73]. These data demonstrated significant relationships between toxoplasmosis-anaemia and toxoplasmosis-hypertension. Congenital toxoplasmosis infection was also detected whereby the rate of serologic evidence in cord blood samples was approximately 0.8%.

Various studies were done to determine the seroprevalence of *T. gondii* infection and associated demographic, clinical, and behavioral risk factors in pregnant women attending antenatal clinic (ANC) at Kilimanjaro Christian Medical Center (KCMC) [74, 75].

In another study involving thirty-seven public hospitals, the mortality patterns of toxoplasmosis and its comorbidities in Tanzania were determined. A 10-year retrospective hospital-based survey was conducted. A total of 188 deaths due to toxoplasmosis were reported during the 10-year period whereby toxoplasmosis deaths accounted for 0.08% (188/247,976) of the total deaths recorded [76].

Livestock farming is important in Tanzania for economic purposes as well as a source of food. Therefore, food-borne parasitic diseases, such as toxoplasmosis, pose a serious threat to economic well-being and food. A cross-sectional study was conducted to investigate seroprevalence and risk factors of *T. gondii* seropositivity in apparently healthy, unvaccinated dairy goat flocks reared under mixed smallholders, northern Tanzania [76]. Seroprevalence of *T. gondii* was found to be 19.3 % of goats and 45.17 % of flocks. Risk factors for infection in goats were identified as sex (infection was significantly higher in females than males), crossbreeding, and district where farm is located. In a separate study, *T. gondii* was detected in 13% of 130 randomly selected farms and seroprevalence was 3.6% in a total of 655 cattle [77]. Risk factors for cattle included herd size and type of farming practice. These data suggest that toxoplasmosis may be posing a significant animal and human health risk and consumption livestock products may play a role in the transmission of the disease to humans.

#### 2.10.5. Sudan and Southern Sudan

Results generated from a study conducted over 30 years ago alleged that toxoplasmosis was less common than other parasitic infections in Gezira Province, The Sudan [78]. About 10 years later, seroprevalence of toxoplasmosis in the same province was shown to be 41.7% [79]. Females within child-bearing age (20-49 years) had a significantly higher prevalence rate than males of the same age. The known food habits of the study population suggest ingestion of *T. gondii* cysts in meat is the main mode of transmission of the disease.

A survey of toxoplasmosis was conducted on pregnant women attending antenatal clinics in Khartoum and Omdurman Maternity Hospitals. Results disclosed seroprevalence levels of 34.1%. Risk factors for IgG anti-Toxoplasma seropositivity were southern ethnic origin and consumption of raw meat. Thirty (18.1%) out of 166 women who were IgG anti-Toxoplasma seropositive gave history of intrauterine fetal death, while 31 (9.7%) out of 321 women who were seronegative gave history of intrauterine fetal death [80, 81].

A study was conducted to determine the effect of toxoplasmosis infection on thyroid hormone levels among Sudanese patients in Khartoum State that help in early diagnosis and treatment. The study showed Toxoplasma antibody types in relation to patient gender; especially, 61.1% of IgG was critical to females including pregnant and nonpregnant, and 38.8% of the same type of antibodies within males and 6.6 % of IgM were critical to females including pregnant and nonpregnant. Also 2.2% of the same type of antibodies within males and 54.4% were abnormal thyroid hormones levels [82].

In a study conducted to determine the seroprevalence of *T. gondii* infections in chickens in Sudan, *Toxoplasma gondii* infection was found to be prevalent. Twenty-five, seventeen, and sixteen chicken sera from Nile River, Khartoum State, and Sennar State were positive, respectively. These represented a seroprevalence of 100% in all states with different titers [83].

Surveys for *Toxoplasma* antibodies in cattle, sheep, goats, and camels (*Camelus dromedarius*) have been conducted [84]. Prevalence was as high as 25% in camels which presents a significant risk for human infection especially among the nomads who consume cameline milk and liver raw.

In another survey carried out to study the seprevalence of *Toxoplasma gondii* in cattle in Khartoum and Gazira States (Sudan), 181 sera samples were collected from dairy cattle with reproductive problems and assayed for antibodies to *T. gondii*. An overall prevalence of *T. gondii* at an individual level in both states was 13.3% (24/181). The prevalence was 12.7% (17/134) and 14.9% (7/47) in Khartoum and Gazira States, respectively [85].

#### 2.10.6. Rwanda and Burundi

Information on toxoplasmosis from Rwanda and Burundi is limited. In Rwanda, the burden and risk factors of *T. gondii* infection among pregnant women and among HIV-infected pregnant women are largely unknown. A study was conducted to determine the seroprevalence of *T. gondii* infections and their risk factors among pregnant women in Kigali, Rwanda. An overall *T. gondii* seroprevalence of 12.2% was determined. Of the 384 pregnant women studied, 37 (9.6%) were positive for anti-*T. gondii*-specific IgG antibodies, indicating past infection and 15 (3.9%) had positive IgM results indicating recent infection [86].

The only recorded seroepidemiological survey for toxoplasmosis in Burundi was conducted in 1985 [87]. The global prevalence among 622 Burundians tested was 44.1% with a higher rate in men (49.6%) than in women (39.2%, *P* < 0.01). Rural areas had higher prevalence than urban areas. These data suggested that toxoplasmic infection is mainly due to oocyst ingestion associated with low levels of hygiene and to a lesser extent to meat consumption in men.

Toxoplasmosis prevalence levels ranging from 12 % to 31% were recorded for two rural populations of Rwanda. *Toxoplasma gondii* was identified as a causative agent in AIDS patients with nervous system complications [88]. Another study was conducted to determine the prevalence of *T. gondii* infections and their risk factors among pregnant women attending antenatal care at Omdurman friendship hospital, Omdurman. Out of the 300 surveyed pregnant women, 65 (21.7%) serum samples were found to have IgG while IgM was showed only 3 (1%) IgM clinics in Kigali, Rwanda.

#### 2.10.7. Eritrea, Djibouti, and Somalia

Toxoplasmosis is an ignored zoonotic disease in the Horn of Africa. After conducting an extensive search, only three studies were found to address the disease in this region. Two surveys conducted over 20 years ago showed that a prevalence exceeding 50% in Somalia while. A survey carried out on 108 abattoir workers of Djibouti slaughter house revealed a prevalence of 42.6% for toxoplasmosis [89, 90].

### 2.11. Ethical Considerations

This a literature review article summarizing observations from several scientific papers that were published in journals requiring that the involvement of human and animals in research be conducted in accordance with relevant national and international ethical guidelines and that the research protocols be approved by the authors' institutional and relevant ethics committee.

### 2.12. Conclusions

Toxoplasmosis is an important zoonosis because it has been detected in one-third of the human population and all types of livestock studies. The infection may very well be a public health disaster-in-waiting and yet remains largely ignored in the Eastern Africa region. Fortunately, the Government of Kenya (GoK) has begun to show interest and established a Zoonotic Diseases Unit (ZDU). The ZDU is a collaborative undertaking between the Ministry of Public Health and Ministry of Livestock Development. It is encouraging that members constituting authorship of the present paper have received financial support from GoK to conduct extensive studies on toxoplasmosis. Currently, these studies are well underway. It is the intention of our research team to be a key resource group for toxoplasmosis in Eastern Africa. We hope to establish and nurture partnerships with like-minded teams around the world.
